# Mitochondrial neurogastrointestinal encephalopathy: a clinicopathological mimic of Crohn’s disease

**DOI:** 10.1186/s12876-018-0925-5

**Published:** 2019-01-15

**Authors:** Ravi Patel, Lucia Lee Coulter, Joanna Rimmer, Miles Parkes, Patrick Francis Chinnery, Oscar Swift

**Affiliations:** 1 School of Clinical Medicine, University of Cambridge, Cambridge, CB2 0SP UK; 2 Department of Gastroenterology, Addenbrooke’s Hospital, Cambridge University Hospitals NHS Foundation Trust, Cambridge, CB2 0QQ UK; 3 MRC Mitochondrial Biology Unit, University of Cambridge, Wellcome Trust / MRC Building, Cambridge Biomedical Campus Hills Road, Cambridge, CB2 0XY UK; 4 Department of Clinical Neurosciences, University of Cambridge, Biomedical Campus, Cambridge, CB2 0QQ UK; 5 Addenbrooke’s Hospital, Cambridge University Hospitals NHS Foundation Trust, Cambridge, CB2 0QQ UK

**Keywords:** Crohn’s disease, Mitochondrial neurogastrointestinal encephalopathy, Azathioprine, Inflammatory bowel disease, Inherited mitochondrial disorders, Thymidine phosphorylase, Leukoencephalopathy

## Abstract

**Background:**

Mitochondrial neurogastrointestinal encephalopathy (MNGIE), due to mutations in *TYMP,* often presents with gastrointestinal symptoms. Two sisters, initially managed for Crohn’s disease based upon clinical, imaging and pathological findings, were later found to have MNGIE. The cases provide novel clinicopathological insight, for two further reasons: both sisters remain ambulant and in employment in their late 20s and 30s; diagnosis in one sister was made after a suspected azathioprine-precipitated acute illness.

**Case presentation:**

A 25-year-old female presented with diarrhoea, vomiting, abdominal pain, and bloating. Faecal calprotectin, colonic biopsies and magnetic resonance enterography were consistent with a diagnosis of Crohn’s disease. Azathioprine initiation preceded admission with a sore throat, headache, myalgia, and pyrexia. Withdrawal led to rapid clinical improvement. MRI brain revealed persistent, extensive white matter changes. Elevated plasma and urine thymidine and deoxyuridine, and genetic testing for *TYMP* variants, confirmed MNGIE. Testing of the patient’s sister, also diagnosed with Crohn’s disease, revealed identical variants. In this context, retrospective review of colonic biopsies identified histological findings suggestive of MNGIE.

**Conclusions:**

Azathioprine interference in nucleic acid metabolism may interact with the mitochondrial DNA depletion of MNGIE. Nucleotide supplementation, proposed for treatment by manipulating mitochondrial nucleoside pools, may require caution. The late onset and mild phenotype observed confirms presentation can occur later in life, and may reflect residual thymidine phosphorylase activity. Clinicians should consider measuring plasma thymidine levels in suspected Crohn’s disease to rule out MNGIE, particularly if white matter abnormalities are identified on neuroimaging.

## Background

Mitochondrial neurogastrointestinal encephalopathy (MNGIE) is an autosomal recessive mitochondrial disorder caused by mutations in *TYMP* leading to reduced thymidine phosphorylase activity and high thymidine levels [1]. Multisystem sequelae include severe gastrointestinal dysmotility [2], as well as cachexia, ptosis, ophthalmoparesis, peripheral neuropathy and leukoencephalopathy. Symptoms typically present before age 20, and untreated, progress relentlessly, with high mortality between the ages of 20 and 40 [3].

All information about the two cases was extracted retrospectively from clinical records. The UK IBD Genetics Consortium dataset of 2513 patients with Crohn’s disease who have undergone whole genome sequencing, was used within a focused, retrospective search [4].

## Case presentation

A 27-year-old white-British female (Patient 1) born to non-consanguineous parents developed intermittent episodes of diarrhea, vomiting and abdominal cramps aged 18, associated with significant weight loss. Symptoms were initially presumed to be post-infectious, given her extensive travel history to Sub-Saharan Africa, South America and South-East Asia. This first episode resolved spontaneously.

**Fig. 1:**
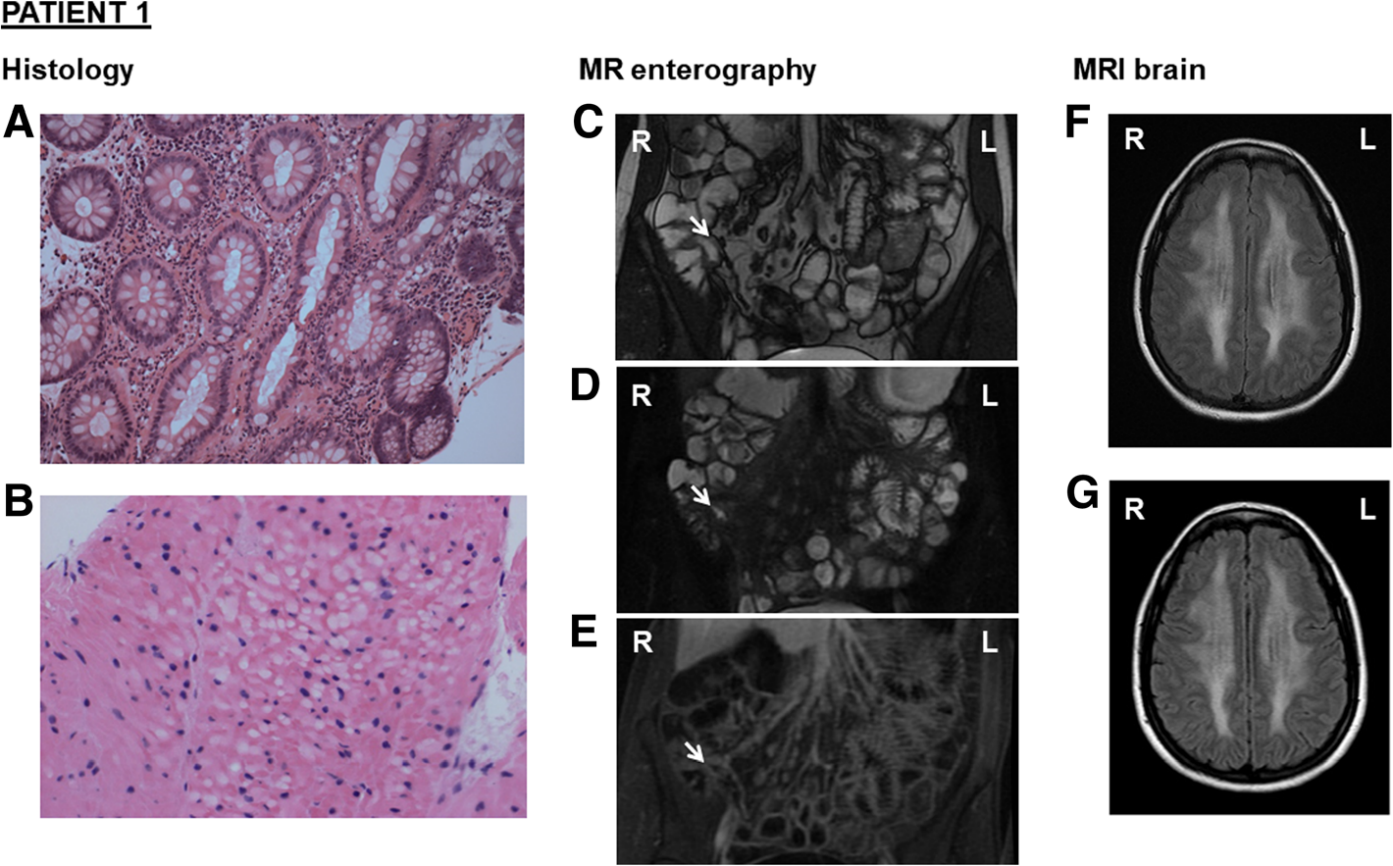
*Colonic histology, MR enterography, and MRI brain for Patient 1 (****a****-****g****).* (**a**) Colonic biopsy showing active inflammation in keeping with panproctocolitis, H&E stain, magnification × 40. (**b**) Colonic biopsy showing focal atrophic changes within the smooth muscle cells of the muscularis mucosa, including intracytoplasmic vacuoles, pyknotic nuclei and eosinophilic degeneration of the cytoplasm, H&E stain, magnification × 200. (**b**-**e**) Coronal contrast MRI of the small bowel demonstrating a 2 cm segment of terminal ileum with mild mural thickening (**c**), mural oedema on T2 sequence (**d**), intermediate enhancement with gadolinium (**e**), and separation of bowel loops in the distal ileal mesentery suggesting fat proliferation. (**f**) Axial brain flair MRI taken during the acute admission, showing extensive confluent, symmetrical white matter hyperintensity in both cerebral hemispheres, which does not show restricted diffusion. This extends from the periventricular to the subcortical regions but sparing the U-fibres. (**g**) Follow-up axial brain flair MRI with contrast, at six weeks post-discharge, showing no change in the extent of diffuse periventricular and subcortical white matter hyperintensity

Aged 25, she represented with diarrhoea, vomiting, abdominal pain and bloating. Family history was notable for an elder sister (Patient 2) with a diagnosis of Crohn’s disease. Faecal calprotectin was significantly raised (> 600; ref. 0–50⎧g/g). Tissue transglutaminase IgA antibody, serial stool samples for faecal culture, ova, cysts and parasites, and *Clostridium Difficile* toxin testing, were negative. Gastroscopy revealed appearances consistent with a dilated second part of the duodenum in keeping with possible small bowel obstruction. Colonoscopy was challenging due to a sharply angulated sigmoid colon and significant patient discomfort. Despite multiple attempts, it was not possible to intubate and biopsy the terminal ileum. Colonic biopsies obtained showed a panproctocolitis compatible with inflammatory bowel disease (IBD) (Fig. 1a). Magnetic resonance (MR) enterography identified a 2 cm segment of terminal ileum with mural thickening and oedema and intermediate enhancement. There was separation of bowel loops in the distal ileal mesentery suggestive of fat proliferation. The remainder of the small bowel and large bowel were normal, with no enlarged nodes, free fluid or fluid collection (Fig. 1c-e).

A diagnosis of Crohn’s disease was made based on clinical, radiological and pathological findings. Treatment with budesonide and, subsequently, azathioprine was initiated. Four weeks after commencing azathioprine, the patient developed sore throat, headache, myalgia, and pyrexia of 41.1 °C.

On examination, she was of short stature with generalized sarcopaenia. BMI was 16.5 kg/m [2]. Cardiovascular, respiratory and abdominal examinations were normal with no extraintestinal manifestations of IBD. Higher cognitive function was normal. Cranial nerve, upper and lower limb, and cerebellar examinations were all normal except for depressed reflexes.

She was treated for presumed meningoencephalitis with empirical broad-spectrum intravenous antibiotics and aciclovir, which were stopped following receipt of sterile blood, urine and cerebrospinal fluid cultures. Echocardiography and CT chest/abdomen/pelvis were unremarkable. Withdrawal of azathioprine during admission led to rapid clinical improvement and apyrexia.

Persistent headaches during the episode led to an MRI brain examination (Fig. 1f), revealing extensive white matter changes that persisted six weeks after stopping azathioprine (Fig. 1g). Subsequent metabolic investigations identified elevated plasma lactate (5.2 mM), plasma ammonia (56 mM), plasma thymidine (10⎧M) and deoxyuridine (15⎧M), and elevated urine thymidine (0.228 mM) and deoxyuridine (0.203 mM), consistent with thymidine phosphorylase deficiency. Genetic testing revealed two heterozygous *TYMP* variants: c.401C > A p.(Ala134Glu) [reported previously [3]] and c.845G > A p.(Gly282Asp) [novel variant], confirming diagnosis of MNGIE. Two years after stopping azathioprine, the patient is well with no active neurological or gastrointestinal symptoms.

**Fig. 2:**
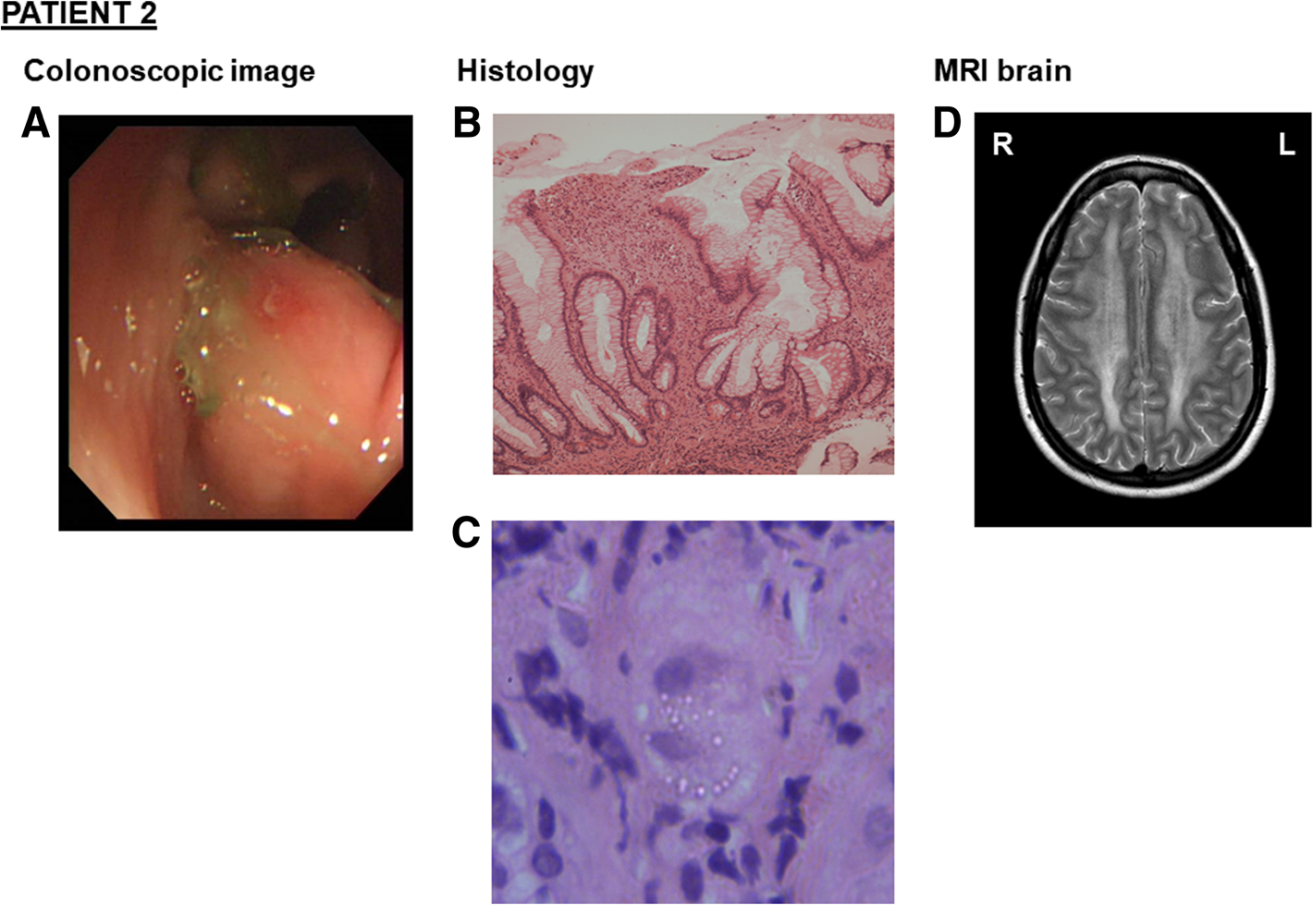
*Colonoscopic image, colonic histology, and MRI brain for Patient 2 (A-F).* (**a**) Colonoscopy showing aphthous ulceration in the descending colon. (**b**) Colonic biopsy showing patchy increase in chronic inflammatory cells within the lamina propria and focal cryptitis, H&E stain, magnification × 40. (**c**) Colonic biopsy showing multiple, round and refractile eosinophilic cytoplasmic inclusions of megalomitochondria within the submucosal ganglionic cells, H&E stain, magnification × 400. (**d**) Axial brain TSE MRI for Patient 2 with white matter changes, as described for Patient 1 Fig. 1**f** and **g**

Her elder sister (Patient 2) had a longstanding history of diarrhoea with nocturnal symptoms, lethargy, loud borborygmi after eating and difficulty maintaining weight. She too had been diagnosed with Crohn’s disease aged 34 following a colonoscopy that had identified aphthous ulceration in her descending colon (Fig. 2a) and biopsy findings consistent with inflammatory bowel disease (Fig. 2b). She had also been treated with budesonide and azathioprine with no improvement in her symptoms. Additionally, she had undergone brain MRI as a healthy control subject for a clinical trial, which identified an incidental leukoencephalopathy (Fig. 2d). Examination at age 38 revealed bilateral ptosis, reduced upwards gaze and lateral eye movements, generalized sarcopaenia, a mild waddling gait and modified Gowers’ manoeuvre. Speech and cognition were normal. Peripheral tone and power were normal. She was areflexic. There were no cerebellar signs. Her BMI was 14.9 kg/m [2].

Subsequent investigations identified elevated plasma thymidine (15⎧M) and deoxyuridine (21⎧M) and elevated urine thymidine (0.6 mM) and deoxyuridine (0.634 mM). Genetic testing identified the presence of the same two TYMP variants c.401C > A p.(Ala134Glu) and c.845G > A p.(Gly282Asp). In the context of having identified TYMP variants, and the common absence of specific histological features of Crohn’s disease, a further retrospective review of colonic biopsies from both patients identified features also suggestive of MNGIE [5] (Fig. 1b, 2c).

## Discussion and conclusions

The family described are notable for three reasons. Firstly, both sisters initially had a working diagnosis of Crohn’s disease. Secondly, both sisters remain ambulant and in employment in their late 20s and 30s, which may reflect that the heterozygous *TYMP* variants identified in these cases manifest as milder forms of the disease. To date, both patients remain clinically stable under supportive management, although allogeneic stem cell transplantation has been discussed as a potential treatment should they deteriorate in the future. Thirdly, diagnosis of MNGIE in Patient 1 was made after an acute illness, possibly precipitated by initiation of azathioprine.

A previous publication identified three MNGIE patients out of a cohort of 102 who had been initially given a working diagnosis of Crohn’s disease [3]. We searched the UK IBD Genetics Consortium dataset of 2513 patients with Crohn’s disease who have undergone whole genome sequencing, for evidence of patients in this cohort who might have been mis-diagnosed as having Crohn’s disease where the true diagnosis was MNGIE [4]. None of these subjects had homozygous or compound heterozygous loss of function mutations in *TYMP*, nor was there evidence of association between this locus and Crohn’s disease susceptibility. The previously reported whole genome sequencing of Crohn’s disease produced median coverage 4x genome-wide [4]. At this sequencing depth it is not possible to exclude ultra-rare variants in *TYMP* in the 2513 patients who were analysed. This would require high coverage sequencing. Their absence does, however, make it unlikely that *TYMP* variation is a significant cause of Crohn’s disease or that MNGIE phenocopying Crohn’s disease is a common problem.

MNGIE has also recently been reported as a mimic of refractory coeliac disease [6], a consideration in the differential diagnosis of non-response to a gluten free diet.

The late onset and mild phenotype observed in these cases may reflect residual thymidine phosphorylase activity and indicates that MNGIE may present later in life than previously recognized [3].

**Fig. 3:**
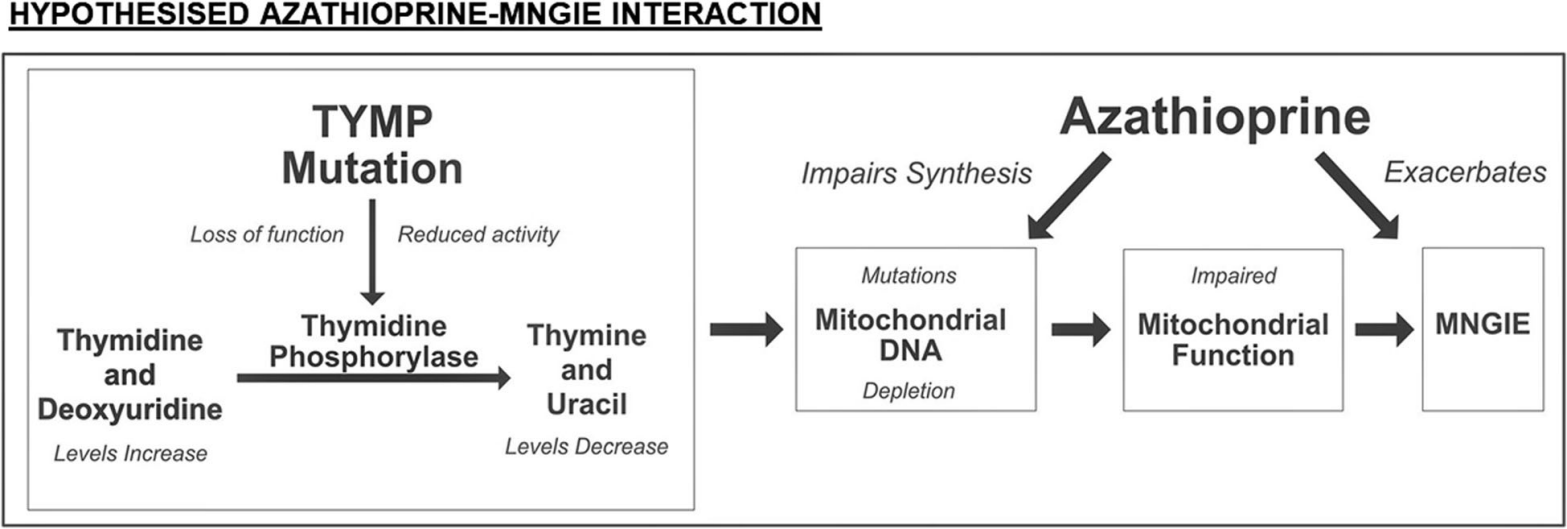
*Schematic of hypothesised MNGIE-azathioprine interaction for Patient 1.* Schematic summarising the hypothesised interaction of azathioprine with the underlying genetic defect of MNGIE, thus potentially explaining Patient 1’s acute deterioration

Clinical features of MNGIE are an indirect consequence of elevated circulating thymidine. This affects intra-mitochondrial nucleoside levels leading to mitochondrial DNA (mtDNA) depletion and secondary mtDNA mutations [7]. Whilst it is recognised that febrile hypersensitivity reactions to azathioprine are not uncommon, it is postulated that azathioprine may further compromise mtDNA maintenance in MNGIE (Fig. 3) through its action as antimetabolite purine analogue [8]. Patient 1’s clinical deterioration was reversible on cessation of azathioprine, possibly reflecting a temporary exacerbation of mitochondrial impairment rather than formation of mtDNA mutations and subsequent cell death.

Alongside haemodialysis, platelet transfusion and allogeneic stem cell transplantation, liver transplantation and nucleotide supplementation have been proposed as treatments for MNGIE [9]. Concern over high mortality rates following allogeneic stem cell transplantation has led to the emergence of liver transplantation as a potential alternative, with high level of expression of thymidine phosphorylase in the transplanted liver shown to normalise the plasma thymidine and deoxyuridine levels [10, 11]. Of these proposed treatments, Patient 1’s case suggests that nucleotide supplementation may require caution because manipulating mitochondrial nucleoside pools could have unanticipated effects. Further investigation into the potential association of *TYMP* mutations with febrile hypersensitivity reactions secondary to azathioprine is needed.

It is important to consider measuring plasma thymidine levels in cases of Crohn’s disease, particularly those with white matter abnormalities identified on brain MRI, which can accompany IBD [12].

## Abbreviations

IBD: inflammatory bowel disease
MNGIE: mitochondrial neurogastrointestinal encephalopathy
MR: Magnetic Resonance
mtDNA: mitochondrial deoxyribonucleic acid
TYMP: thymidine phosphorylase
